# Fleas infesting pets in the era of emerging extra-intestinal nematodes

**DOI:** 10.1186/1756-3305-6-59

**Published:** 2013-03-07

**Authors:** Donato Traversa

**Affiliations:** 1 Department of Comparative Biomedical Sciences, University of Teramo, Teramo, Italy

## Abstract

Modifications in climatic conditions, movements of hosts and goods, changes in animal phenology and human behaviour and increase of wildlife, are presently concurring in the geographic spread of vectors and cardio-respiratory nematodes, e.g. *Dirofilaria immitis*, *Angiostrongylus vasorum*, *Aelurostrongylus abstrusus* and *Capillaria aerophila*. All these factors may also influence dispersion and clinical significance of fleas, thus posing relevant challenges in those regions where other parasites are emerging at the same time. *Ctenocephalides felis*, *Ctenocephalides canis* and *Pulex irritans* cause discomfort, nuisance, allergic reactions, anaemia, and may transmit several pathogens, some of them are of importance for public health. The present article reviews the importance of fleas in small animal practice and their sanitary relevance for dogs, cats and humans, and discusses current control methods in the present era of emerging extra-intestinal nematodes, towards a possible changing perspective for controlling key parasites affecting companion animals.

## Review

### Background

Cardio-respiratory nematodes affecting dogs and cats are nowadays prevalent in several countries, where they have a growing importance due to their clinical impact, possible zoonotic hazard, and geographical emergence and spreading in both endemic regions and areas previously free of infection. This is the case for the mosquito-borne *Dirofilaria immitis*, the mollusc-borne *Angiostrongylus vasorum* and *Aelurostrongylus abstrusus*, and for *Capillaria aerophila*, which has a direct biological cycle, albeit earthworms may have an unclear role of facultative intermediate or paratenic hosts [1-4]. Modifications in epidemiological patterns and clinical approaches for these infections are changing perspective and perception on parasitoses of pets and could lead to the fallacy that other parasites of dogs and cats present nothing new to be investigated and disseminated. This potential mistake with “old-fashioned” parasites has been recently discussed for roundworms, hookworms and whipworms [5,6]. This could be true also for ectoparasites like fleas, although they have a major pathogenic role in human and veterinary medicine. Fleas are a cause of direct damage to the hosts’ skin, they are hated by pets and owners for the nuisance and distress they cause, and have a powerful vectorial ability.

**Figure 1:**
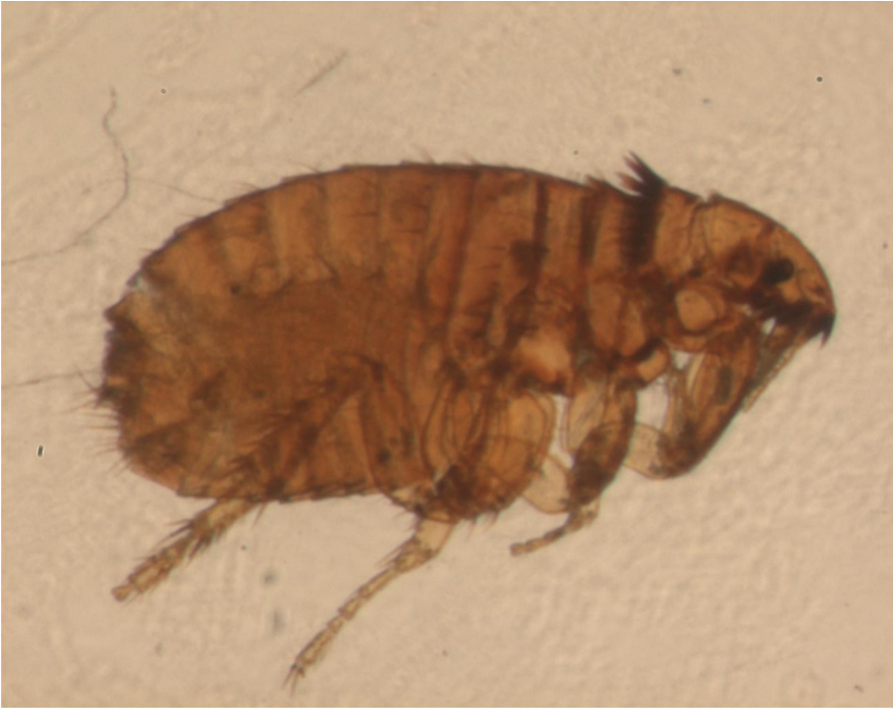
**Adult *Ctenocephalides felis*.**

**Figure 2:**
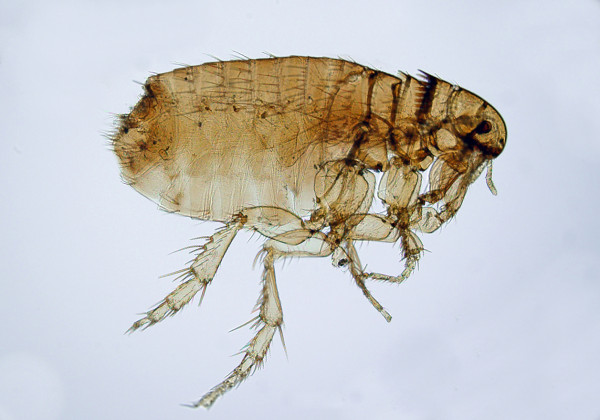
**Adult *Ctenocephalides canis.***

**Figure 3:**
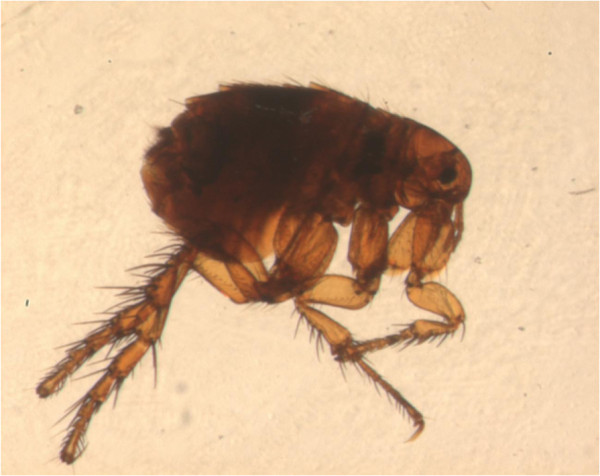
**Adult *Pulex irritans*.**

Of the more than 2,500 known fleas, three major species feed on pets and humans [7-9]. The most widespread is the cat flea *Ctenocephalides felis* (Figure 1), which is highly prevalent in both dogs and cats in all corners of the world. Also the dog flea *Ctenocephalides canis* (Figure 2) and the human flea *Pulex irritans* (Figure 3) are globally distributed, although with lower rates [9,10]. These fleas have a low degree of species-specificity, being able to infest companion animals, humans and wildlife. Canine and feline pulicosis is characterized by high infection rates everywhere [7,9,11-14], thus treatment and prevention are a priority in veterinary medicine. However, the control of fleas is not straightforward and requires integrated approaches. Infected pets and environments are still a major cause of striving for veterinarians and pet owners, although a plethora of safe and effective products is available to be used either on the animal or in the environment, or both.

A shift of perspective on pet parasites is occurring in those regions where epidemiological patterns of cardio-pulmonary nematodes are changing. In particular, veterinarians are often faced with the necessity of treatment and prevention approaches controlling at the same time both common, e.g. fleas and intestinal worms, and emerging, e.g. *A*. *vasorum* and *A*. *abstrusus*, parasites. Hence, the present review aims to provide food for thought on control methods for fleas of pets in the present changing era of emerging heartworms and lungworms. Sanitary importance of fleas, their possible spreading, and integrated control strategies are discussed in a comprehensive perspective.

### Fleas: a threat for dogs, cats and humans

Fleas are primary ectoparasites causing discomfort, allergic manifestations and anaemia, and having an efficient vectorial ability for various pathogens. The present section summarizes the most important pathogenic aspects of *C*. *felis*, *C*. *canis* and *P*. *irritans*.

**Figure 4:**
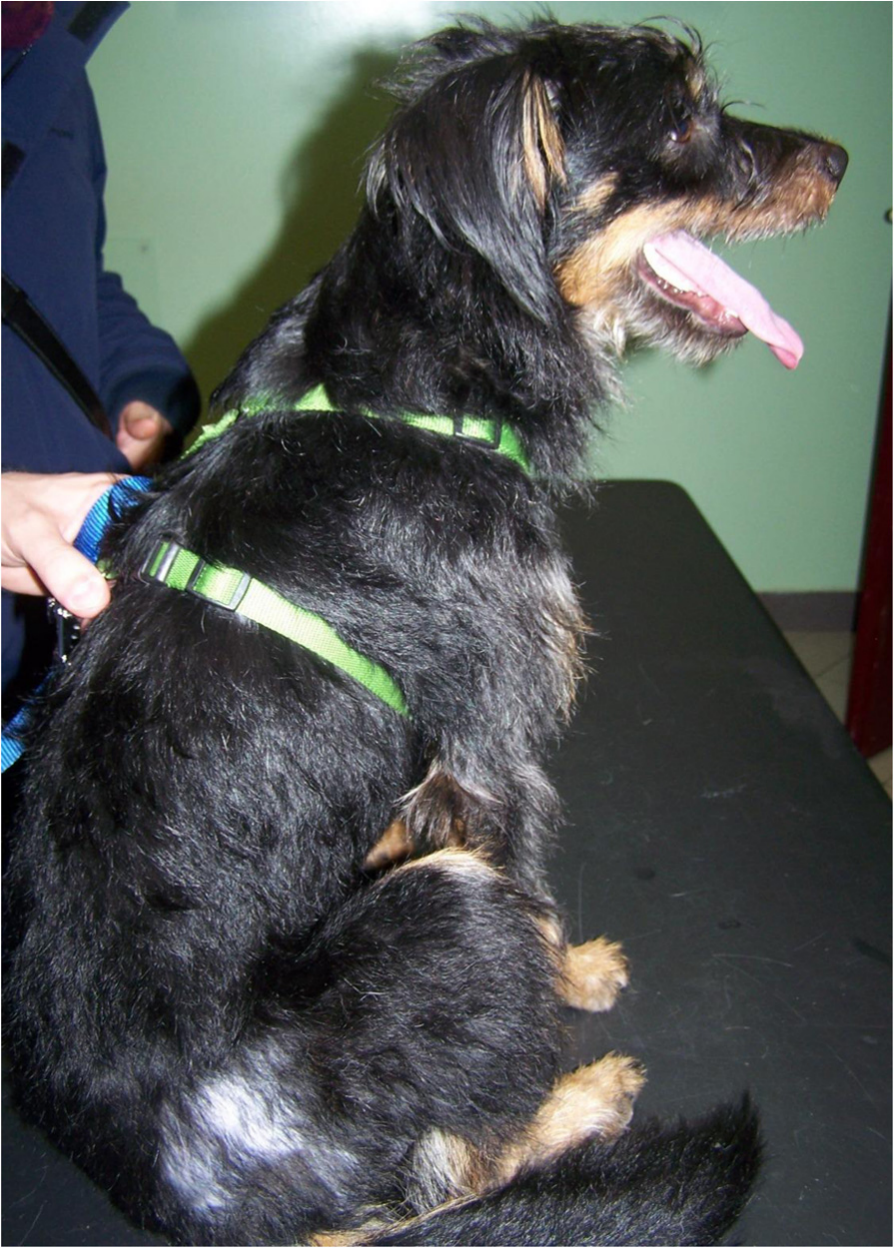
Localized alopecia in a dog with flea allergic dermatitis.

**Figure 5:**
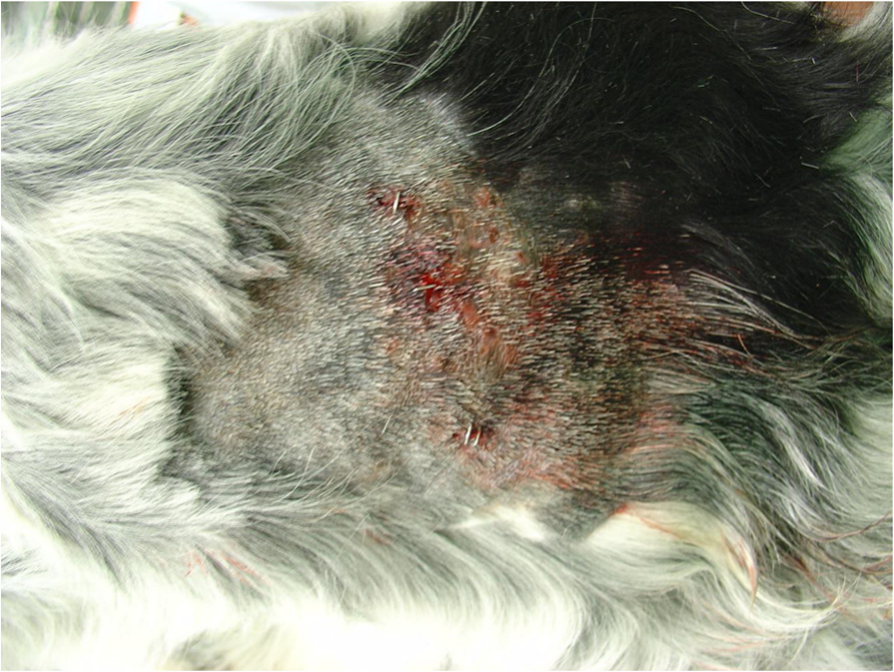
Alopecia and self-trauma induced by biting and scratching in a dog with flea allergic dermatitis.

**Figure 6:**
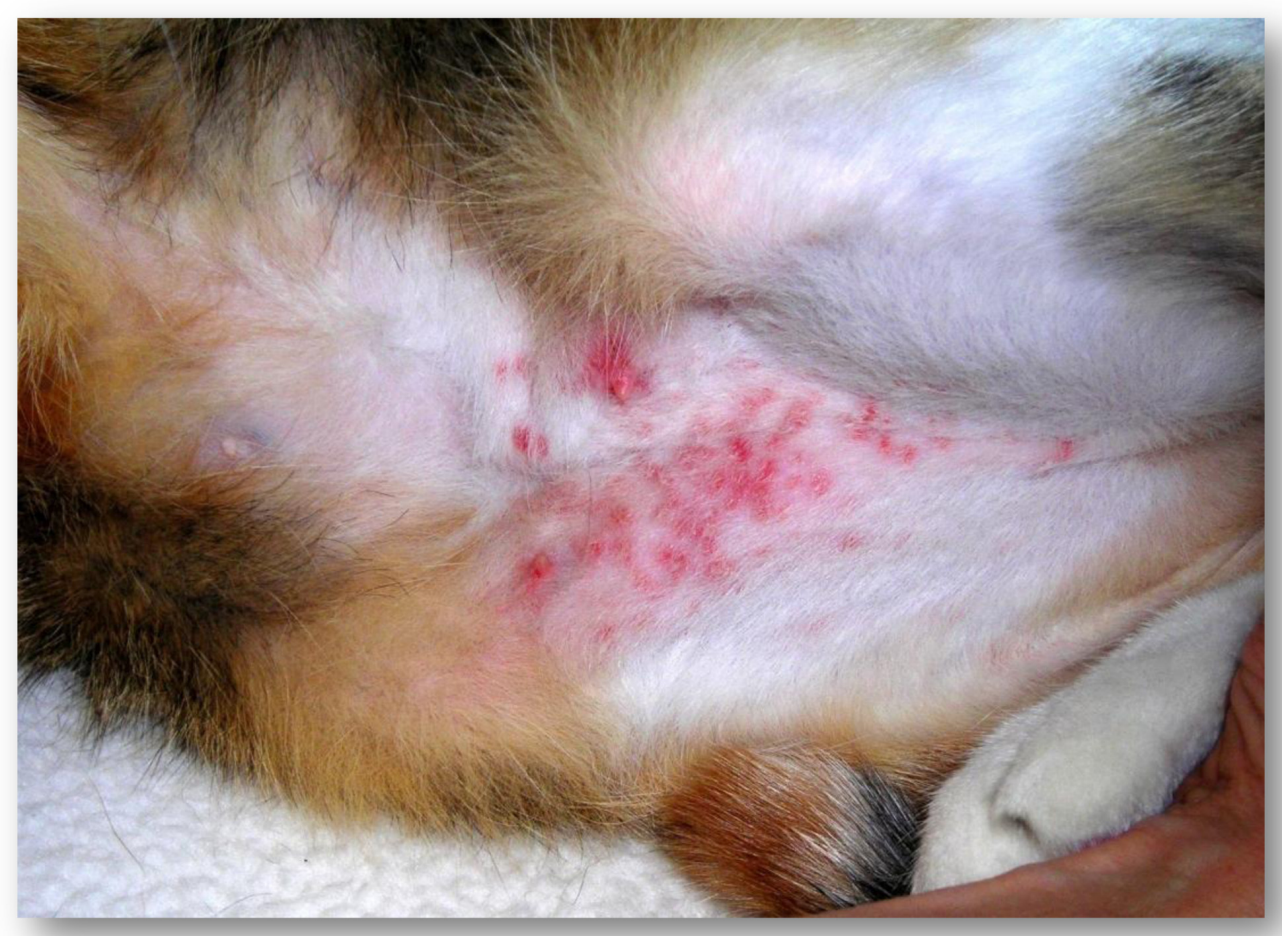
Flea allergic dermatitis in a cat.

**Figure 7:**
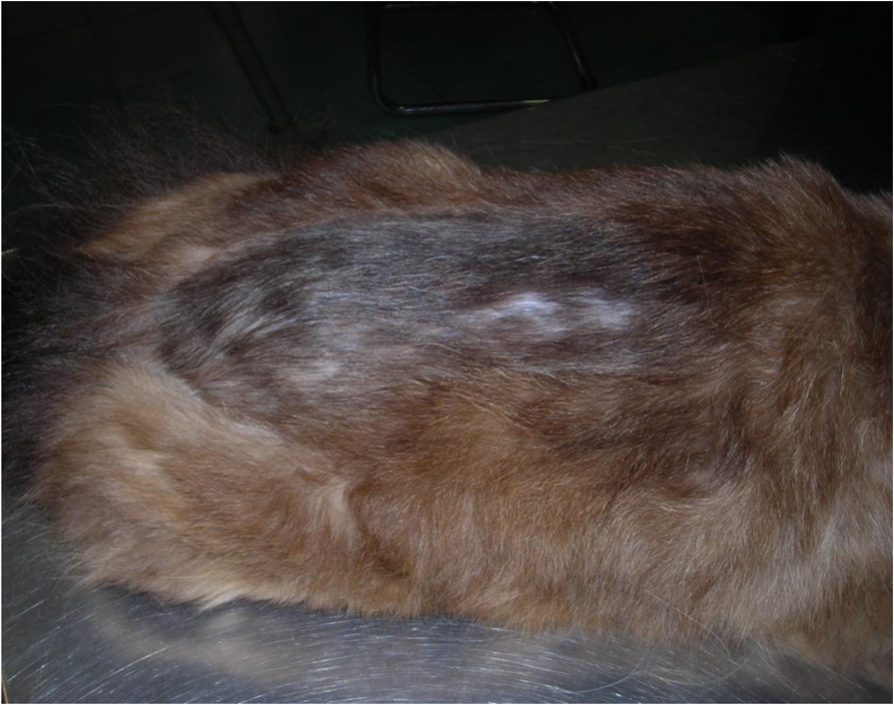
Localized alopecia in a cat with flea allergic dermatitis in a cat.

Biting of adult fleas is followed by a delayed reaction and skin irritation. The lesions initially appear as single or clustered small haemorrhagic areas. Thereby, a wheal forms around each bite, with a sudden peak in few minutes and, most often, the onset of itching. The lesion may become a hard papillar lesion [10]. Repeated exposure to flea bites induces, in susceptible dogs and cats, a condition called flea allergy dermatitis (FAD). Dogs with FAD (Figure 4) present with erythema, alopecia, excoriation, papules, crusts, itching often leading to self-traumas (Figure 5), while cats show a miliary dermatitis (Figure 6) with nibbling, alopecia (Figure 7), intense pruritus, licking, scratching, self-traumas [15-18]. Along with other allergic diseases (e.g. food allergy diseases), FAD is a major clinical entity in pets, one of the most important skin conditions of household animals, and one of the most frequent causes for seeking veterinary advice [15,19].

Blood-feeding adults of *Ctenocephalides* spp. may cause iron deficiency anaemia especially in puppies and kittens [15,20]. In the case of long-lasting infections, also adult animals may suffer of blood loss and chronic anaemia [15,20].

The plague-causing bacterium *Yersinia pestis* circulates in rodent populations mainly through bites of the “oriental rat flea” *Xenopsilla cheopis*, which may also feed on pets and human beings. Cats are a potential source of plague, while dogs appear to be less susceptible to *Y*. *pestis*[9,21]. Although a correlation between human plague and sleeping in the same bed as a dog has been documented [22], the ability of *Ctenocephalides* spp. bites in transmitting *Y*. *pestis* is considered low [23]. *Ctenocephalides* spp. are only occasionally found on rodents and considered unlikely bridging vectors of plague between animals and humans [23-25]. Therefore, long and close contact with pets and their fleas is necessary for the transmission of plague from dogs to humans via their fleas [26]. The role of *P*. *irritans* as a vector of plague has yet to be elucidated [27], even though this species has been collected from rodents within established plague foci and it has been considered a driver for human-to-human transmission of the disease [24,28].

*Ctenocephalides felis* can be naturally and experimentally infected with *Rickettsia typhi*, the causative agent of the “murine typhus”, a zoonotic disease which circulates in rodents via the oriental rat flea [29]. *Pulex irritans* has also been experimentally proved to be a vector of *R*. *typhi*, although considered unlikely to be a primary source of the infection [30]. Cats and dogs have been found seropositive for *R*. *typhi* in both Europe and USA [31,32], thus they might have a certain epidemiological role in the dispersion of this zoonosis [33]. *Rickettsia felis*, a pathogen mainly transmitted by the cat flea [34] and suspected to induce a murine typhus-like illness in humans, is worldwide distributed, due to the global dispersion of *C*. *felis*, the number of infected cats and the possibility of transstadial and transovarial transmission in fleas [35-37]. *Ctenocephalides canis* and *P*. *irritans* may also act as vectors of *R*. *felis*, and both cats and dogs have been found harbouring this bacterium, although their epidemiological role is yet to be fully addressed [30,37-39].

The so-called human “cat-scratch disease” is mainly caused by *Bartonella henselae* and *Bartonella clarridgeiae*. Cats are their reservoirs, while dogs may be accidentally infected by *Bartonella* spp., among which *Bartonellavinsonii* subsp. *berkhoffii* is incriminated as a cause of a human cardiac disease in tropical areas [40,41]. While cats are recognized hosts for animal and zoonotic bartonelloses, the role of dogs as a source of human infections is not clearly established [42,43]. Fleas of pets are capable of harboring *Bartonella* spp. and it seems plausible that *C*. *felis* transmits *B*. *henselae* actively. The human infection by flea-borne *Bartonella* spp. generally occurs via scratches of infected cats, whilst flea bites do not seem to play a key role in the epidemiology of human bartonellosis. Infection of cats by bites of infected fleas has been experimentally demonstrated, although not considered a primary route of transmission [30,44,45].

Fleas of pets are competent intermediate hosts of the tapeworm *Dipylidium caninum*. This zoonotic cestode is the most prevalent in dogs and cats and is often associated with pulicosis in pets, stray animals, kenneled dogs and cats in colonies. Animals and humans become infected by accidentally ingesting residues or whole fleas containing the infectious cysticercoid. Indeed, *D*. *caninum* may cause disease especially in children with low hygiene standards [9,15].

*Ctenocephalides felis*, *C*. *canis* and *P*. *irritans* are intermediate hosts for the filarial nematode *Acanthocheilonema reconditum*, transmitted via their bites to dogs and human beings and a cause of subcutaneous infection in animals and ocular disease in humans [46-48].

### Drivers nurturing the epidemiology of cardio-respiratory nematodes and the dispersion of fleas: analogies and differences

Environmental conditions, host availability and preferences, abundance and feeding behavior of vectors and hosts are the most important drivers influencing the geographic dissemination of helminths and arthropods [2,49,50]. Additionally, the epidemiology of vector-borne parasites is associated with vector phenology and biology, movements of goods and animals, globalization, increase of wildlife in peri-urban and urban areas, and climate changes [2,3,51].

Global warming promotes survival and reproduction rate of arthropods and molluscs. Consequently, it may nurture their dispersion, abundance, intensity and temporal patterns throughout the year. At the same time, climate might influence flea development and distribution, along with rates of disease transmission in urban, suburban and rural areas. Although warmer temperatures predicted in future climatic scenarios could lead to an increased expansion of fleas, especially into the Northern hemisphere [52], data proving whether and how global warming is a driving force for fleas and transmitted diseases needs to be definitively corroborated, although evidence is growing.

The use of the Geographic Information System (GIS) and predictive models provides information for studying the epidemiology of vectors and transmitted pathogens [53,54]. Warmer climates and higher air temperatures favour insect breeding and may reduce parasitic extrinsic incubation periods [2,55], as shown for *D*. *immitis*[2]. Analogously, *A*. *vasorum* is expanding, and it is expected to increase its distribution in European regions which offer ecological and epidemiological conditions for the expansion of current endemic areas and the establishment of further new endemic foci [56]. At the moment no computer modelling is available for the emerging respiratory nematode of cats transmitted by snails and slugs, i.e. *A*. *abstrusus*. However, geographic spread of gastropods, nurtured by climate changes, may play a role in the incidence and distribution of all mollusc-borne parasitoses [57,58], including aelurostrongylosis. *A. vasorum* and *A*. *abstrusus* have similar life cycles, thus the same drivers implicated in the spread of *A*. *vasorum* would likely also have an effect on *A*. *abstrusus* in the same areas.

Current environmental modifications could influence fleas. Thus geographical regions where cardio-pulmonary parasites are spreading will be probably faced also with changes in flea distribution and patterns of infestation. Fleas are ectothermic arthropods susceptible to fluctuations of temperatures, especially because immature stages live outside the host. Indeed, climatic variations have an impact on fleas and flea-borne diseases because their epidemiology results from the biological interactions between hosts, pathogens and vectors, which are individually influenced by temperature, humidity and precipitation.

Larval stages and pupae live within the living environment of animals, while adult fleas are permanent parasites, feeding on their hosts. The speed of juvenile flea development depends on the environment, in that higher temperatures may increase the number of generations and colder temperatures and higher humidity has an effect on the longevity of fleas in the absence of available hosts [9]. In particular, the higher the environmental temperature, the higher the rate of development, until a critical value is reached. Beyond this value, survival of immature stages decreases, especially in the presence of low humidity [52]. Temperatures >35°C and <3°C, in combination with a relative humidity <33%, may impair flea development [59-61]. Accordingly, the vast majority of past plague cases have been described in those regions where average temperatures are >13°C, with outbreaks occurring when temperature ranges from 24°C to 27°C and a decrease of epidemic activities at higher values [62-64]. Additionally, rainfall favours vegetative production, increase of small-mammal populations, rat flea-infestation rates, along with soil moisture, which promotes flea survival rates [65,66]. This scenario enhances the probability of contact between infectious fleas and susceptible hosts and, consequently, of epizootic cases of plague [30,67]. As an example, epizootics in prairie dogs from Montana, USA, were shown to be positively associated with precipitation and warm days, but negatively associated with hotter days [68].

Given that warm and moist weather are factors beneficial for flea development, microclimatic drivers like temperature, rainfall, relative humidity and climate changes in general, are expected to largely modify the future dispersion of plague and of flea-borne disease [69]. Studies have shown that fleas have a seasonality in both the Northern and Southern Hemispheres, starting their activity in spring or increasing from a low base during spring and peaking in mid to late summer followed by a decline until winter [14,70-72]. Until about fifteen years ago, cat fleas were considered to be rarely found on pets in cold seasons [73], but the present weather changes characterized by shorter winters [74,75] may have a potential effect on flea activity in the near future. This is particularly true because short winters permit survival of fleas, especially in the stage of quiescent adults inside the cocoon [73]. It is interesting to note a recent study from Germany, which indicated the absence of a relationship between seasons, climatic conditions and presence of pulicosis in dogs and cats. Above-average warm weather in summer 2003 was an apparent cause of doubling documented flea infections, probably because of outdoor environmental conditions favourable for flea development. The highest infestation rates were detected in July-October and July-September for dogs and cats, respectively. However, fleas were also present on animals in the remaining months of the year although with lower rates. In this study, no statistical differences were found in infestation patterns between the four seasons, albeit prevalence of pulicosis in dogs and cats was higher in the summer [11].

More attention is required to understand to what extent climatic conditions will affect the distribution of flea and diseases in different epidemiological settings [69]. Hence, worthy of mention is the “FleaTickRisk” model, designed in collaboration between climatologists, biomathematicians and parasitologists, able to predict, on a weekly basis, the activity and abundance of cat fleas (and three tick species) in Europe, and the risk of disease transmission [76]. Feeding behaviour and availability of suitable hosts are other key factors impacting on the distribution of emerging nematodes and, possibly, of fleas.

Destruction and/or reduction of natural habitats oblige wild animals to move into new hospitable environments, in the suburbs and cities. The dispersion of wildlife (e.g. feral cats, hedgehogs, opossums, raccoons, foxes, rodents) increases the spreading of their pests and pathogens, often shared with humans and pets [77,78]. The presence of red foxes (*Vulpes vulpes*) in cities and peri-urban areas is likely concurring in causing the emergence of heartworms and lungworms [1,3,79-82]. Red foxes are common reservoirs for *D*. *immitis* in several European countries [1], with prevalence up to ~25-30% in Spain and Italy [79,82]. The current trend in the higher incidence of canine angiostrongylosis and the concomitant expansion in foxes demonstrates that the parasite is emerging in wild reservoirs, along with establishment and spreading in companion dogs [4,56]. Finally, the recent finding of genetic haplotypes of *C*. *aerophila* shared between foxes, beech martens, cats and dogs in European countries, ultimately supports the existence of common patterns of transmission for respiratory nematodes between wildlife and pets [83]. Interestingly, the most recent published case of human lung capillariosis has been recently described from Serbia, where the infection rate of *C*. *aerophila* in foxes is very high [84,85].

Given that flea populations are shared among domestic and wild reservoirs, household dogs and cats could be more frequently exposed to pulicosis, as for extra-intestinal nematodes. In fact, abundance of suitable hosts, and attractiveness and tolerance of wild animals to flea bites, influence the survival and development of fleas. Most fleas are generalist rather than specialist, thus this ability makes wildlife a source for pulicosis for pets and humans and bridging hosts for transmitted pathogens. Fleas infesting feral animals may be picked up by privately owned pets and brought into homes, thus becoming a nuisance and potentially carrying pathogens of human and veterinary concern [86]. Indeed, wildlife represents an “overwintering strategy” for fleas of pets, because mammals living in sub-urban and urban territories may be infested by fleas throughout the year, thus they are a source of infestation for cats and dogs, especially in spring and summer [73,87]. Repeated reports of *C*. *felis*, *C*. *canis* and *P*. *irritans* in wild animals, e.g. red foxes [88-90], confirm that they serve a source of maintenance. Altogether, the susceptibility of both pets and wildlife to the same species of fleas, lungworms and heartworms, suggests that epidemiological interactions play a crucial role in spreading infections in endemic and areas previously free of infection, when suitable bridging epidemiological settings occur. These scenarios have a major relevance also for the dispersion of flea-borne pathogens, as shown by the key examples from the following.

Human dwellings in African regions endemic for plague are considerably infested by the human-associated fleas *C*. *felis* and *X*. *cheopis*, other than *P*. *irritans*[27,30]. Outbreaks of murine typhus in the USA have been attributed to the presence of infected cats and opossums, the high number of opossums infested by *C*. *felis*, the maintenance of *R*. *typhi* in the cat flea/opossum cycle and the avidity of cat fleas in biting humans [35,37,91-93]. A similar situation has been described for *R*. *felis*, as shown by its presence in *C*. *felis* collected from opossums in the USA [86]. Recently, *B*. *henselae* and *B*. *clarridgeiae* were detected in cat fleas from *V*. *vulpes* in Australia and *B*. *clarridgeiae* in the blood of a fox, thus suggesting that this wild canid may act as a reservoir of bartonellosis for fleas, animals and humans [94]. A study in the USA showed antibody titres for *Bartonella* spp. in three different felids, including feral cats, living in sympatry along urban edges and with degrees of contact with each other, home range and resource requirements [95].

Indeed, data on the natural cycle of flea-borne pathogens in wildlife from Europe are scanty. For example, *B*. *henselae* has been recently found in a wildcat (*Felis silvestris*) and *R*. *felis* was found in *C*. *felis* infesting wild animals in Spain [96,97]. Also, the DNA of *Bartonella* spp. and *R*. *felis* has been found in fleas biting a wood mouse in the Netherlands [98] and in wild animals from Germany [99]. Seropositivity for *R*. *typhi* in different species of wild rodents has also been detected in Spain [100].

### Fleas and cardio-pulmonary nematodes control: many more things in common than we probably think

Treatment and prevention of fleas are necessary to resolve sanitary and pest problems associated with the infestation of pets and homes, and to minimize the exposure to transmitted diseases. To effectively eradicate a flea infestation, both adult and immature stages must be eliminated. This appears simple but owners and veterinarians struggle daily with fleas, because this mission can be accomplished only with reliable and integrated approaches, avoiding one-off methods.

The administration of insecticides on an animal infested with existing fleas permits control of adult stages. However, the vast majority of the flea populations live for a long time outside the host, thus the environment is a key factor to treat pulicosis and prevent re-infections [7,101,102]. When an infested dog or cat moves around, thousands of eggs may roll off from the hair onto baskets, beds, carpets, blankets, sofas, where immatures may survive for months.

Control of fleas in the environment can be obtained by administering parasiticide formulations acting on both adult and immature stages to the animal, or by using adulticides along with combinations containing chemicals effective against environmental juveniles. Direct measures may integrate the use of chemicals but they are not useful alone [103]. These measures could rely on insecticidal dusts to treat runways, burrows, pet bedding and premises in general. Regular vacuum cleaning of carpets, blankets and pet bedding areas reduces the contamination by flea eggs.

Prevention in animals free of fleas may be achieved also by residual insecticides alone and/or repellents, but they are out of the scope of the present review and have been discussed elsewhere [7,104]. This section will focus only on those chemicals useful to treat and/or prevent fleas, which are available in combination with compounds used in the control of cardio-pulmonary nematodes of dogs and cats. Below are provided examples of studies carried out to evaluate their efficacy in controlling flea infestations.

Macrocyclic lactones (MLs) belonging to the group of avermectins/milbemycins are endectocides with a broad spectrum of activity, including nematodes and several arthropods. They have a GABA-mimetic effect via the binding of glutamate-gated chloride channels. MLs may be available in combination with insecticides/acaricides to expand their spectrum of activity, including fleas.

Among these drugs, topical selamectin kills adult *C*. *felis* and prevents flea eggs from hatching. Selamectin eliminates up to 98.3% and 90.1% of newly acquired adult fleas within 24 hours on days 21 and 28 after treatment, respectively, 99.0% of fleas within 48 hours from the infestation at 28 days after treatment, and it may allow control of 97.3% adult fleas at 31 days after treatment [105,106]. Selamectin reduces egg production, has ovicidal effects impairing larval emergence from eggs in days 2–37 post-treatment (hatch rate 0–13.2%) and allows a 23.3% larval emergence rate at Day 45 post treatment [106]. It has been suggested that debris contaminated with selamectin kill eggs and larvae probably by contact and ingestion, leading to ~98% decrease of egg production and ~92% failing in egg hatching [107,108]. This drug can be used on a monthly basis to treat existing pulicosis and prevent the development of fleas in environmental conditions [109].

Neonicotinoids, spinosins and insect growth regulators (IGRs) are common parasiticides present in formulations also containing MLs licensed for the control of cardio-pulmonary nematodes.

Neonicotinoids cause spastic paralysis of insects with an agonistic effect on postsynaptic nicotinic acetylcholine receptors of motoneurons. Imidacloprid, one of the most used of these chloronicotinyl compounds in both dogs and cats, has a residual activity lasting about a month [104]. Its efficacy relies on contact with fleas, which die within 24 hours and sometimes between 2 and 8 hours after infestation [104,105,110]. Other than a quick adulticidal activity against already existing infections on pets, imidacloprid presents a significant flea larvicidal effect [111-113]. Single topical applications of imidacloprid provide control and reduction of flea numbers of > ~95-97% on cats and dogs for 28–37 days, and >98.6% reduction of off-host stages in premises [114-116]. After application on the hair of a pet, sufficient amounts of imidacloprid are transferred to the surrounding environment (e.g. blanket) preventing a high percentage of larvae from developing into adults at about 1 month [113]. Larvae in the pet’s living environment are killed after contact with a treated dog or a cat, resulting in the reduction of developing fleas and in preventing (re-)infestation of treated animals for at least 4 weeks (e.g. highly satisfactory 36-hours flea kill rate at 27–41 days after treatment), even though with a varying degree of decrease in the speed of kill (e.g. at Day 28 72.6% of the fleas killed within 48 hours of infection, or at Day 34 90.8% flea reduction at 24 hours) throughout the month following application [105,112,113,117-120]. Other than alone, this insecticide is available in combination with ivermectin (e.g. in North America) and moxidectin.

Spinosins bind nicotinic acetylcholine receptors of insects, causing a stimulation of post-synaptic neurons [104]. The efficacy is based on contact or after ingestion, thus they are marketed in topical or oral formulation. The compound consisting of a mixture of spinosins A and D, i.e. spinosad, has an anti-flea efficacy starting half an hour after oral administration and reaching 100% efficacy at 4 through 48 h post-treatment against existing infestations in dogs [121]. Its persistent efficacy against re-infestations has been shown to last about one month, although with a certain degree of reduction in speed of action (i.e. speed at 4 hours of 74% and 42% after 3 and 4 weeks) and in killing efficacy against *Ctenocephalides* spp. (i.e. 100% at 24 hours during three weeks, then decreasing until 85% and 80.4% at Day 28 and Day 43 respectively) [121-124]. Spinosad, moreover, is effective in reducing flea egg production (>99.8% for about 1 month) although its absence in the skin debris of treated dogs results in no effects on environmental flea stages [121]. Other than alone, this chemical is also available, at the moment only in North America, in combination with milbemycin oxime.

IGRs inhibit the reproduction in adult insects and block the organogenesis of immature instars via hormonal or enzymatic influence. Of the two groups of IGRs available, i.e. juvenile hormone analogs and chitin synthesis inhibitors, molecules belonging to the latter group are available in formulations containing also MLs. Chitin synthesis inhibitors have effects both on adult stages on the animal and on immature fleas in the environment. Female adult fleas suffer from a reduction of prolificacy and fecundity, while egg hatching and larval moults are inhibited [104]. Lufenuron, belonging to the group of benzoyl-phenyl-ureas (BPUs) and classified as an insect development inhibitor (IDI), is available either in injectable or oral formulations, the latter also containing milbemycine oxime. Lufenuron has ovicidal and larvicidal activity but no adulticidal effect [125,126]. This molecule provides flea control on the animal and in the environment because female fleas biting treated animals will generate eggs from which no larvae will hatch and juveniles feeding on pre-digested blood in the environment are unable to moult to the next stage [125,127,128]. A three-year study demonstrated that the monthly administration *per os* of lufenuron to cats and dogs is able to provide long-term reliable control of fleas [78]. Lufenuron proved to be highly effective when used alone in simulated home environments with induced flea infestations [129-131], but a fast acting adulticide should be also used if a flea infestation is already established at the beginning of the treatment [132]. A 3-months trial showed that the combined administration of oral lufenuron once a month, and of an insecticide at two different regimens, may reduce flea populations on pets by at least 97.3% within one week, maintaining this level of reduction for 90 days, along with a highly effective control level for off-host stages [133]. Indeed, the concomitant use of adulticides greatly supports the elimination of adult fleas, especially within the first 3 weeks of therapy, which is crucial especially in situations with intense flea challenge [134]. A recent 90-day study carried out with the oral combination containing lufenuron and milbemycin oxime showed that no adult stages are generated from eggs laid by fleas present on experimentally infected dogs which are treated 30 days apart for three times [135].

Apart from selamectin, the aforementioned compounds are available in formulations also containing MLs potentially useful to control cardio-pulmonary nematodes.

A spot-on containing imidacloprid 10%/moxidectin 2.5% can be used for preventing canine angiostrongylosis for its effective and safe larvicidal activity in dogs experimentally infected with *A*. *vasorum*. A single topical application is 100% effective in the elimination of fourth stage larvae (L4) and pre-adults of the parasite, thus preventing patent infections. A monthly-based use of this topical spot-on potentially prevents or, at least, minimizes the establishment of adult *A*. *vasorum*, the severe cardiopulmonary tissue damage caused by the parasite and the clinical onset of the disease [136]. Milbemycin oxime is another potential option for preventing canine angiostrongylosis because an efficacy of 85% has been reported in dogs, which received 0.5 mg/kg of the drug at 30 and 60 days after experimental infection with *A*. *vasorum*[137].

MLs can be used to prevent canine heartworm disease in both dogs and cats for their ability to kill third and fourth larval stages of *D*. *immitis*[138]. Milbemycin oxime and moxidectin can be administered in dogs once a month during the season of activity of mosquitoes, with the first dose given within a month after the beginning of the season and the last dose within 1 month after vectors disappear [138,139]. The spot-on formulation containing imidacloprid 10%/moxidectin 1% and oral milbemicyn oxime may be used also for the prevention of *D*. *immitis* infection in cats [140-143]. As an example, the imidacloprid 10%/moxidectin 1% spot-on provides 88.4-100% control of adult *C*. *felis* for 35 days in cats, other than treatment and control of intestinal nematodes and heartworm [143,144]. When milbemycin oxime is associated with praziquantel the formulation can be used also for tapeworms, and when associated with lufenuron the combination is effective also against immature fleas [5,6]. Topical selamectin can be used to prevent cardio-pulmonary dirofilariosis in dogs and cats and, as mentioned above, this formulation is active against fleas and other major parasites of companion animals [107,138,139].

### May control of fleas support prevention of cardio-pulmonary nematodes and *vice versa*?

In newly acquired infestations, the first fleas jumping and biting on a pet are unseen and the animal remains untreated for several days or weeks [102]. The infestation is instead noticed when the animal has become parasitized by a high number of fleas. At that time, the untreated animal has already dispersed flea eggs in the environment, which have given rise to larvae, pupae, and newly emerging adult fleas. This results in an infestation diagnosed only when the home is already contaminated with hundreds to thousands of flea life stages, potentially causing continuous re-infestations for the pet [102,145,146].

The ideal goal is a pet free of fleas throughout the year, regardless an existing pulicosis, and protected even when it comes in contact with these ectoparasites. In general, a year round approach is not applied for fleas by pet owners and veterinarians, because control of pulicosis is traditionally suggested from late winter or early spring and continued throughout summer until autumn. This is usually based on seasonal treatment with formulations containing residual insecticides and/or repellents [7,17,18,101,147], most of which are out of the scope of the present article. In any case, collars are not usually replaced more than once a year, and those with the longest activity do not encompass 12 months; moreover, spot-on and spray formulations are usually not applied monthly for a year by the owners. Hence, all pets can be at risk of exposure to fleas in certain months. This is particularly true if one considers that differences may not exist in flea infestation patterns between the four seasons of the year [11]. As an example, although multicentre field trials have successfully demonstrated the monthly efficacy of spinosad in naturally infected dogs during the summer, i.e. the period of highest level of contamination for homes [148], control of fleas is not imperatively seasonal.

The duration of chemoprevention for parasites affecting pets is often questioned and, for instance, the US Companion Animal Parasite Council (CAPC) and the European Scientific Counsel Companion Animal Parasites (ESCCAP), have different guidelines. In particular, there has been a debate on the duration of the monthly chemoprophylaxis for *D*. *immitis*, i.e. if all year round, six months, or only during the mosquito season [149-151]. Indeed, the availability of parasiticide formulations providing monthly protection from *D*. *immitis* when mosquitoes are active and continued control of intestinal infection by nematodes, could make the interruption of an all year round control program undesirable. This is particularly true where the risk of heartworm transmission is considered low but pets may be infected with intestinal nematodes (e.g. whipworms, hookworms, roundworms) throughout the year [5,6,149]. Hence, the CAPC suggests to use broad-spectrum parasiticides year round to have a pet free of intestinal worms, along with the prevention for *D*. *immitis*. Such an approach could be endorsed by veterinarians and pet owners elsewhere because, regardless of the ubiquitous distribution of intestinal worms [5,6], cardio-pulmonary nematodes are spreading and/or emerging in several countries of Europe [3,152], and there is a risk of importation and establishment (*A*. *vasorum*) in the Americas [153]. The ESCCAP has suggested extending chemoprevention for *D*. *immitis* to 7–8 months or even year round [2], given that certain vectors (e.g. the Asian tiger mosquito *Aedes albopictus*) may survive in temperate areas as adult stages, even during winter [154]. This mosquito has the potential to extend animal and human risk of exposure to heartworms during the whole year, especially in urban habitats of southern areas [2]. Also, large-scale and prolonged preventative measures against canine dirofilariosis may promote a decrease in the prevalence in those dogs living in the same area, but not subjected to the prophylaxis. In fact, chemopreventatives reduce the abundance of reservoirs, as shown in some areas of Northern Italy [155], thus they could be useful in reducing the spread of *D*. *immitis* also where the infection has a low prevalence and/or the nematode has emerged recently [2,156].

Another important example is provided by the French heartworm *A*. *vasorum*, which is expanding in several countries of Europe where it may overlap the distribution of *D*. *immitis* and even cause co-infections [3,157]. Additionally, this nematode also represents a potential threat for North America, e.g. through imported infected hosts [153]. Hence, there is the actual risk that veterinarians will need to use chemopreventative approaches with MLs also for canine angiostrongylosis which, at present, is suggested in those areas with high epidemiological hazard [3,136].

In summary, several parasiticides used for the control of intestinal nematodes and chemoprevention of *D*. *immitis* and, possibly, of *A*. *vasorum*, contain chemicals useful in flea control programs. They add further support to the yearly use of some products in particularly risky situations, given that domestic temperatures allow ectoparasites (e.g. fleas) to survive, develop and infect pets (and sometimes owners) throughout the year [149]. Therefore, the all-year round treatment with broad spectrum combinations can be applied to assure treatment, prevention and/or control of major parasites, including intestinal and cardio-pulmonary nematodes (and other parasites, according to the formulation), especially when immature stages of fleas should be kept under control in the home environment.

### Final remarks

Fleas are a constant danger for animal and human health for the primary pathogenic potential they have and for the pathogens they transmit with their blood meals or faeces. Thus, there is a constant concern over their control in most countries of the world.

Apparent evidence that the present climate changes and reduction in the length of winter season could interfere in the biology of fleas in the near future is growing. Of importance, is that fleas may survive indoors during the winter and also outdoors in those situations in which the environment is marginal [158]. For instance, epidemiological changes in fleas and transmitted diseases are suggested by the apparent emergence of bartonellosis in Europe [159,160]. Also detection of *R*. *felis* in fleas is increasing in a number of countries, thus the distribution of this pathogen is nowadays considered as wide as the globally distributed vector *C*. *felis*[76].

At the same time, these epidemiological changes could contribute to “new” sanitary problems especially where intestinal worms are endemic and cardio-respiratory nematodes are spreading. Flea-borne transmitted pathogens have been found in fleas collected in countries where heartworms and lungworms are endemic or emerging (see ref [3] for geographical spread of cardio-pulmonary nematodes in Europe). As key examples, both *B*. *henselae* and *B*. *clarridgeiae* have been found in fleas collected from dogs and cats in France and Germany [161], Albania [162], Spain [90], the Netherlands [98], Hungary [163], UK [159], and in the USA as well [164]. In Italy, *Bartonella* spp. is endemic especially in the South [165,166], where human seropositivity to cat scratch disease has been unveiled [167]. Moreover, *R*. *felis* has been found in cat fleas collected from companion animals throughout the country [13,168]. At the same time Italy is a key country for the emergence and spreading of cardio-pulmonary nematodes, with *D*. *immitis* being hyper-endemic in the North and expanding southward, *A*. *vasorum* potentially spreading in all the territory, and *C*. *aerophila* and *A*. *abstrusus* widely distributed with prevalence rates up to 7% and 20% respectively [3,56,152,169,170]. Considering that in Italy, and possibly in other countries, intermediate and paratenic hosts of heartworms and lungworms can be present throughout the year [2,3], the opportunity of all-year-round controls should be taken into account.

Control and prevention of flea infections rely on strategic treatments with persistent adulticidal products (e.g. imidacloprid) and/or with chemicals acting against off-host immature stages (e.g. lufenuron) along with adulticides, if an infestation already pre-exists [7,104,171-174]. In the case of survival of fleas to an adulticide, and consequent production of eggs, IGRs may ultimately prevent the development of larvae [104,175]. This is of importance because the efficacy of a year-long flea control program, e.g. using monthly applications, may have some constraints if based only on the use of residual insecticides, killing primarily adult fleas.

Indeed, different products (e.g. imidacloprid, spinosad, selamectin) have an excellent activity in killing existing adult fleas on pets [104,105,114,133,172,173,176-178]. Interestingly, several studies have evaluated parasiticides against the “KS1 cat flea strain”, which has been maintained at the Kansas State University, USA, since 1990 and has varying levels of resistance or reduced susceptibility to different compounds [146]. However, the susceptibility of this strain to different formulations at varying regimens (e.g. lufenuron, spinosad, imidacloprid + moxidectin, selamectin and others out of the scope of this review) is clear evidence of the efficacy of compounds presently marketed in controlling pulicosis for about an entire month [135,146,179,180]. A rapid action is also critical, because fleas should be killed as soon as they emerge from the cocoon and jump on a pet. Adult *C*. *felis* begins feeding almost immediately once on an animal, with many fleas feeding within minutes [20]. The more quickly a product kills newly acquired fleas, the more effectively it prevents FAD and reduces the likelihood of transmission of pathogens. This is problematic, especially when client compliance with environmental treatments is inconsistent, thus causing recurrent infestations, and when residual efficacy of insecticides declines after the first weeks post-administration [181,182]. Hence, breaking the life cycle of fleas by interruption of their reproduction is crucial. Most residual flea adulticides have a prolonged activity and they either kill or intoxicate newly acquired fleas within one day after re-infection [183]. Larvicidal effects of imidacloprid is of practical significance in both breaking the flea life cycle and reducing the level of flea infestation in the domestic environment, spinosad and selamectin reduce egg production, and the latter has ovicidal effects impairing larval emergence [106,111,112,121]. Given that most insecticides may suffer from decreasing levels of killing speed throughout the month following administration [105,106,123,124], environmental control is mandatory. This is even more important if one considers that rapid and voracious blood sucking by adult fleas (e.g. 25–60% of fleas are blood fed within 5 min and partially digested blood can be defecated in as little as 2–6 minutes after fleas infest a host) may indeed impair prevention of flea biting and feeding by residual insecticides [101,184,185]. This aspect could also have practical implications in clinical management of FAD. A single flea is generally considered enough to elicit and maintain the clinical signs of FAD [186], but such ability has been recently questioned. If this dogma were true, no flea product would provide high control against FAD, at least not until the flea population is eradicated [101]. This is in contrast with the evidence that residual insecticides (among others selamectin, imidacloprid) have high efficacy in reducing the occurrence of FAD. Therefore, it is suggested that the key drivers of FAD are the degree of hypersensitivity of an individual animal, the number of fleas feeding and amount of salivary antigens injected [101]. Regardless of the role of a single flea in eliciting FAD, the total flea eradication from the environment is necessary.

## Conclusion

In conclusion, new concepts for reliable preventative plans are presently being discussed in the scientific community, with regard to epidemiological modifications in several areas. With ectoparasites, a correct understanding of basic knowledge for the control of fleas and related diseases is important for the selection of appropriate products by veterinarians. There is a constant necessity to update practitioners on the possibilities they have to control fleas affecting pets because they may be not aware on the actual applicability of available formulations, especially in terms of speed of kill of residual insecticides, ability to control feeding and reproduction of fleas, and potential for compounds acting on off-host immature stages [7,101]. Hence, the choice of the control method should always be made according to the concurrent epidemiological risk not only for ubiquitous intestinal nematodes, like *Trichuris vulpis*, but also for emerging heartworms and lungworms and for the “ancient” fleas.

Given the new epidemiological scenarios, separate mono-products with different target parasites would probably be inferior if compared with a single combination able to control all major parasites at the same time, for their a broader spectrum of activity. This is especially true where intestinal worms and fleas are highly endemic and cardio-respiratory nematodes are emerging. The high number of flea products either in combination or as single compounds to be administered in strategic alternations in flea control programmes is also important to minimize the risk of the onset of resistance. In general, blanket administration of broad-spectrum parasiticides should be discouraged, given that the abuse of anthelmintics can promote drug resistance. Actually, at the moment there is only evidence of resistance to pyrantel in canine hookworms. Although pyrantel is not used for monthly prevention of cardio-pulmonary parasites, a high level of attention should always be maintained to detect any hint of resistance to parasiticides, including MLs, in nematodes of pets. [6]. This is even more important if one considers that there is the first laboratory evidence that microfilariae and third-stage larvae of *D*. *immitis* can show a degree, although how much is yet to be established, of resistance to certain parasiticides [187].

On the other hand, further studies are warranted to extend the spectrum of activity of those formulations containing molecules active against fleas and useful to control heartworms and lungworms. For instance, it would be insightful to thoroughly evaluate the efficacy of ivermectin and selamectin against *A*. *vasorum*, *A*. *abstrusus* and *C*. *aerophila* in clinical trials, given the scant reports published thus far [188-190]. The recent reports of the efficacy of the spot-on formulation containing moxidectin in the treatment of feline aelurostrongylosis and capillariosis [169,191] should encourage further studies aiming to also investigate the applicability of chemopreventative approaches against these parasites, as recently assessed for dog angiostrongylosis [136]. Also, given the established efficacy of milbemycin oxime against *A*. *vasorum*[137] it could be important to evaluate its activity in treating and preventing *C*. *aerophila* in dogs and cats and *A*. *abstrusus* in cats.

## Competing interest

All scientific aspects of the present article have not been influenced by any third party.

## Author contribution

DT conceived the intellectual content of the article and wrote the text.
